# Isolation and Characterization of Nanocrystalline Cellulose Isolated from Pineapple Crown Leaf Fiber Agricultural Wastes Using Acid Hydrolysis

**DOI:** 10.3390/polym13234188

**Published:** 2021-11-30

**Authors:** Fitriani Fitriani, Sri Aprilia, Nasrul Arahman, Muhammad Roil Bilad, Amri Amin, Nurul Huda, Jumardi Roslan

**Affiliations:** 1 Doctoral Program, School of Engineering, Post Graduate Program, Universitas Syiah Kuala, Banda Aceh 23111, Indonesia; fitriani18@mhs.unsyiah.ac.id; 2 Department of Chemical Engineering, Universitas Syiah Kuala, Banda Aceh 23111, Indonesia; nasrular@unsyiah.ac.id; 3 Faculty of Integrated Technologies, Universiti Brunei Darussalam, Bandar Seri Begawan BE1410, Brunei; roil.bilad@ubd.edu.bn; 4 Department of Mechanical Engineering, Engineering Faculty, University of Abulyatama, Lampoh Keudee, Aceh Besar, Banda Aceh 23372, Indonesia; amriamin@abulyatama.ac.id; 5 Faculty of Food Science and Nutrition, Universiti Malaysia Sabah, Jalan UMS, Kota Kinabalu 88400, Malaysia; jumardi@ums.edu.my

**Keywords:** nanocrystalline cellulose, pineapple crown leaf, thermal properties, morphology properties, acid hydrolysis

## Abstract

Pineapple crown leaf fiber (PCLF) is one of the major biomass wastes from pineapple processing plants. It consists mostly of carbohydrate polymers, such as cellulose, hemicellulose, and lignin. It can be further processed to form a more valuable and widely used nanocrystalline cellulose (NCC). This study investigates the effect of hydrolysis time on the properties of the produced NCC. The acid hydrolysis was conducted using 1 M of sulfuric acid at hydrolysis times of 1–3 h. The resulting NCCs were then characterized by their morphology, functional groups, crystallinity, thermal stability, elemental composition, and production yield. The results show that the NCC products had a rod-like particle structure and possessed a strong cellulose crystalline structure typically found in agricultural fiber-based cellulose. The highest NCC yield was obtained at 79.37% for one hour of hydrolysis. This NCC also displayed a higher decomposition temperature of 176.98 °C. The overall findings suggest that PCLF-derived NCC has attractive properties for a variety of applications.

## 1. Introduction

Pineapple crown leaf fiber (PCLF) is one of the major agricultural wastes generated from pineapple processing plants. The upper crown of a pineapple fruit accounts for approximately 10–25 percent of its total weight [1]. Its processing generates 3 billion tons of byproducts per year, causing an environmental problem in agricultural lands [2]. This biomass waste primarily comprises carbohydrate polymers containing cellulose, hemicellulose, and lignin [1]. Along with these three major components, PCLF also contains minor components such as extractives, inorganic compounds, and ash [1,3,4]. According to previous studies, the content of cellulose, hemicellulose, and others in PCLF was 79–83%, 19%, 5–15%, and 4–5%, respectively [3,5,6,7,8]. As an abundant renewable resource, cellulose can be a feedstock for the production of environmentally friendly and biocompatible derivatives.

Cellulose is a glucan polymer composed of D-glucopyranose units linked by β-1,4-glycosidic bonds [9,10]. Naturally, cellulose resembles microfibrils and consists of crystalline and amorphous regions. The bundles of microfibrils are joined together by a hydrogen bond formed between the hydroxyl groups and oxygen. All native cellulose has been classified as cellulose type I with intra- and intermolecular hydrogen bonds [3].

Nanocrystalline cellulose (NCC) is a non-fibrous, purified, and partially depolymerized form of cellulose. NCC is commercially produced from woody plants and purified cotton. NCC is created by treating cellulose derived from plant materials, most commonly through conventional acid hydrolysis. Generally, NCC has diameters of around 5–30 nm and lengths of 100–500 nm. NCC has appealing properties such as a high reinforcing capability, good thermal stability, biodegradability, and biocompatibility [11,12]. NCC is a crystalline powder that is light in color, odorless, and tasteless [13]. Its structural characteristics, such as particle size, thermal tolerance, and crystal rigidity, are primarily determined by the raw material sources, as well as process conditions such as pretreatment methods, treatment reactions, hydrolysis duration, variety of acid, acid concentration, and temperature [11,14,15,16]. NCC is utilized in the food processing, pharmaceutical, and cosmetic sectors as a value-added product [17]. Nowadays, commercial NCC has various physicochemical, morphological, and thermal properties, resulting in a variety of functional parameters and applications [16,18]. Furthermore, they are widely used as reinforcing fillers in biocomposite production fields.

There are only a few available reports on the extraction and characterization of PCLF-based NCC [1,19]. Prado and Spinace [1] prepared cellulose nanocrystals from PCLF using acid hydrolysis (60% H_2_SO_4_). However, the nanoparticles obtained in this study were only obtained from a specific hydrolysis reaction. There was not an aspect of the study that investigated the impact of the hydrolysis reaction time on the resulting NCC. In our previous work, we reported the effect of the hydrolysis reaction time on the resulting NCC obtained from the PCLF in terms of their physicochemical properties and characteristics [19]. However, the report excluded the thermal and morphological properties of the resulting NCC.

In this study, we investigate the effect of the hydrolysis reaction time on the characteristics of the produced NCC from PCLF. The NCCs were produced under 1–3 h of hydrolysis and characterized in terms of morphology, functional groups, thermal stability, elemental composition, and production yield.

## 2. Materials and Methods

### 2.1. Materials

The PCLF was obtained from the residue of juice and fruit salad produced in a local market in Banda Aceh, Indonesia. Sodium hydroxide (NaOH, Merck, Darmstadt, Germany), hydrogen peroxide (H_2_O_2_, Merck), and sulfuric acid (H_2_SO_4_, Merck) were used as alkali, bleaching, and hydrolysis agents.

### 2.2. Preparation of Nanocrystalline Cellulose from Pineapple Crown Leaf

**Figure 1:**
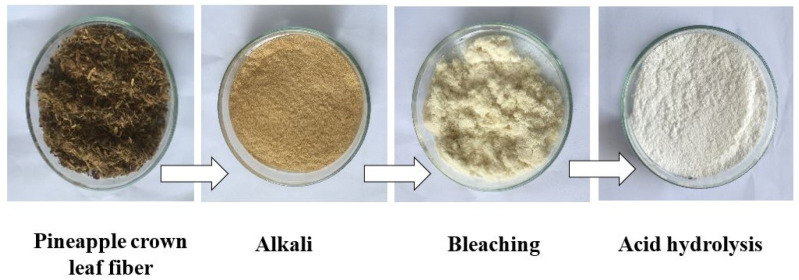
Schematic process of the isolation of nanocrystalline cellulose from pineapple crown leaf.

NCC was obtained from PCLF by pre-chemical treatment according to the method reported previously [19]. Before removing the non-cellulosic component, the PCLF was washed with warm water at a temperature of 40 °C then dried in an oven at 60 °C for 24 h to remove impurities. The dried PCLF was ground into powder form and sieved under 40 mesh. The alkali and bleaching treatments of the PCLF were performed using 1 M of NaOH and 1 M of H_2_O_2_ at 80 °C for 1 h. After the process was completed, the samples were rinsed several times using distillate water until reaching a neutral pH. After the alkali and bleaching treatments, the samples were hydrolyzed using 1 M of H_2_SO_4_ under various reaction times (1, 2, and 3 h) at 45 °C. The acid hydrolysis reaction was stopped by adding distilled water, then cooling in a water bath at 20 °C for 24 h. The NCC suspension was washed using distillate water, followed by centrifugation at 2000 rpm for 30 min and ultrasonication for 30 min. This was done to remove the excess acid until a neutral pH was reached. Finally, the resultant product was dried and ground into powder to obtain the NCC solid product. The NCC samples obtained under various hydrolysis times of 1, 2, and 3 h are labeled as NCC-1, NCC-2, and NCC-3, respectively. The schematic process of the isolation of NCC is illustrated in Figure 1.

### 2.3. Lignocellulosic Composition and Yield Analysis of Nanocrystalline Cellulose

The identification of the lignocellulosic composition of PCLF was carried out according to the Chesson–Datta method [20,21]. The main procedure in this method is to remove extractives, hydrolyze the hemicellulose with a strong acid without heating, then perform dilute acid hydrolysis at high temperatures. A solution containing 1 g of dried sample (a) and 120 mL of distilled water was first heated at temperature 100 °C in the water bath for 1 h. The solution was filtered, and the residue was washed with hot water. The residue was dried in an oven until the weight was constant to produce residue (b). The residue (b) was then dissolved in 150 mL of 1 N H_2_SO_4_ and heated at a temperature of 100 °C in a water bath for 1 h. The solution was subsequently filtered and washed with distilled water, and the residue was dried until the weight was constant (c). The residue (c) was later soaked with 10 mL of 72% H_2_SO_4_ at room temperature in the water bath for 4 h. Then, 150 mL of 1 N H_2_SO_4_ was added to the solution and heated at a temperature of 100 °C in the water bath for 2 h. The final residue was filtered and washed with distilled water until reaching a neutral pH. After that, the residue was heated in the oven at 105 °C until a constant weight was achieved (d). Lastly, the dried residue was heated in the furnace at 600 °C until it formed ash, which was then weighed (e). The percentage of the chemical composition of the PCLF was calculated using Equations (1)–(5).

(1) $$Hot\;water\;soluble\;(\%)=\frac{a-b}{a}\;\times \;100\%\;\;$$

(2) $$Hemicellulose\;(\%)=\frac{b-c}{a}\;\times \;100\%$$

(3) $$Cellulose\;(\%)=\frac{c-d}{a}\;\times \;100\%$$

(4) $$Lignin\;(\%)=\frac{d-e}{a}\;\times \;100\%$$

(5) $$Ash\;(\%)=\frac{e}{a}\;\times \;100\%$$

where *a* (g) is the initial weight of the PCLF sample, *b* (g) is the residue weight at the two weighings, *c* (g) and *d* (g) are the residue weight at the third and fourth weighing, and *e* (g) is the weight of the ash. The percentage yield of each NCC sample’s production was calculated using Equation (6) [11]:

(6) $$Yield\;(\%)=\frac{(m_{2}-m_{3})}{m_{1}}\times 100\%$$

where *m*_1_ is the mass of raw PCLF, *m*_2_ is the total mass after the hydrolysis process within the weighing container, and *m*_3_ is the mass of the weighing container.

### 2.4. Morphology and Thermal Properties of Nanocrystalline Cellulose

#### 2.4.1. Fourier Transform Infrared Spectroscopy

The functional group of the NCC samples was investigated using Fourier transform infrared spectroscopy (FTIR, IRPrestige-21, Shimadzu, Kyoto, Japan) with wavenumbers ranging between 4000 and 400 cm^−1^. The samples were ground with potassium bromide (KBr) and formed a transparent film before the analysis.

#### 2.4.2. Crystalline Analysis

The crystallinity of the NCC samples was investigated by using X-ray diffraction (XRD, XDR 7000, Shimadzu, Kyoto, Japan) with Cu Kα radiation (*λ* = 1.5406 Å) at an operating voltage of 40 kV and 30 mA in the 2*θ* range of 10–80° for scanning. The crystallinity index (*Crl*) was calculated using Segal’s method as shown in Equation (7) [22,23]:

(7) $$Crl\;(\%)=\frac{(I_{200}-Iam)}{I_{200}}\;\times \;100\%$$

where *I*_200_ is the maximum intensity of the diffraction at 200 peaks (2*θ* = 22.5°) and *I_am_* is the minimum intensity of the diffraction at 2*θ* = 18.1°. The crystallite size of the NCC samples was calculated using the Scherrer Equation (8):

(8) $$L=\frac{0.94\;\lambda }{\beta \;cos\theta }\;\times \;100\%$$

where *L* is crystallite size (nm), *λ* is the X-ray wavelength, *β* is the full width half maximum (FWHM, in rad), and *θ* is the corresponding Bragg angle [24].

#### 2.4.3. Scanning Electron Microscopy

The surface morphology of the NCC samples was examined by using scanning electron microscopy (SEM, JSM-6360LA, JEOL Ltd., Tokyo, Japan). The NCC samples were coated with a thin layer of gold to impose conductivity, and the analysis was performed under 1–14 kV of accelerating voltage.

#### 2.4.4. Thermogravimetric Analysis

The thermal properties of the NCC samples were evaluated using thermogravimetric analysis (Shimadzu DTG-60, Kyoto, Japan). About 5 mg of each NCC sample was heated from 25 to 550 °C at a constant heating rate of 10 °C min^−1^ under an inert atmosphere to analyze the thermogravimetric (TGA) and the derivative thermogram (DTG).

## 3. Results and Discussion

### 3.1. Lignocellulosic Composition and Yield of Nanocrystalline Cellulose

Lignin, hemicellulose, and cellulose are the three main components of PCLF biomass. The compositions of each lignocellulosic component contained in the PCLF sample are shown in Table 1. The percentage of cellulose content of 51.2% from the pineapple leaf in this study is not comparable to the research results reported by Santos et al. [4], Cherian et al. [25], or Mamani et al. [26], which were 74.50%, 81.27%, and 56% respectively. This variation can be attributed to the part of the leaf and the type of raw material used, the different locations of collection, and the treatment process or method used to determine the composition [3]. The lignocellulose compositions from the studies of Santos et al. [4], Cherian et al. [25], and Mamani et al. [26] are shown in Table 1.

Table 2 shows the yields of all NCC samples obtained from the PCLF. The breakdown of 1,4-glycosidic chains at prolonged acid hydrolysis duration caused a decrease in the yield of the produced cellulose and degradation in the amorphous part of the cellulose [27,28]. Meanwhile, the crystalline part with a regular chain structure is more resistant to acid treatment [29]. The same results were obtained in a previous study conducted by Aprilia et al. [30]. The increase in hydrolysis time was inversely proportional to the yield of obtained cellulose from kenaf tree bark. The yield in this study was similar to the cellulose nanocrystal yield from pineapple leaves reported earlier by Santos et al. [4] with acid hydrolysis under varying reaction times. The yields obtained in the present study were also higher than the cellulose yield from rice hull and bean hull fibers, which were 52.06% and 75.39%, respectively [31]. The high NCC yields obtained in this study suggest the high potential of PCLF as a source of cellulose.

### 3.2. Characterizations of Morphology and Thermal Properties of Nanocrystalline Cellulose

#### 3.2.1. Fourier Transform Infrared Spectroscopy

**Figure 2:**
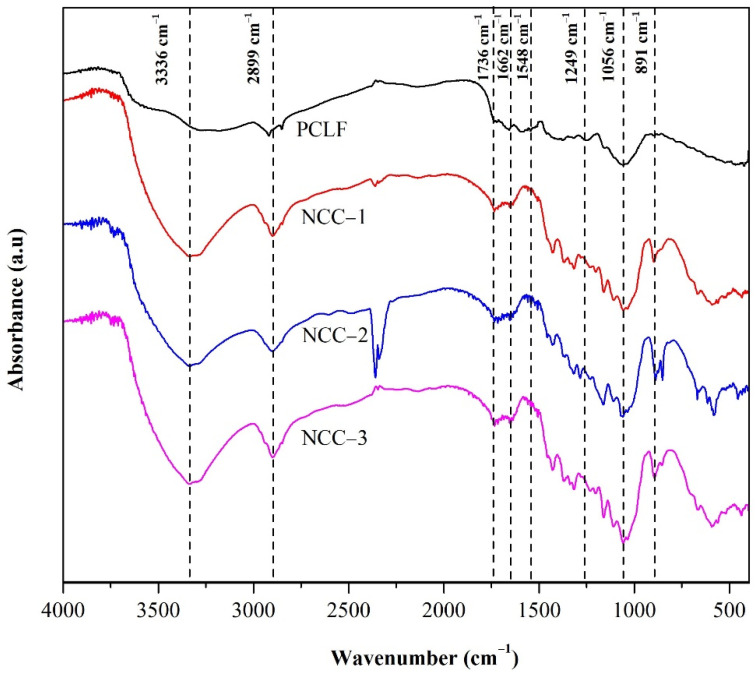
FTIR Spectra of nanocrystalline cellulose from pineapple crown leaf fibers with various hydrolysis times.

The FTIR spectra obtained for the NCC samples from the PCLF with various hydrolysis times are shown in Figure 2. A broad peak in the range of 3500–3200 cm^−1^ is associated with the -OH vibration group of cellulose and intermolecular hydrogen bonding. The presence of hydroxyl groups in the nanofibers caused a deeper peak in this range with increased hydrolysis time, indicating the hydrophilic properties of NCC [32]. The interference of OH from water was eliminated by ensuring all samples were completely dry. The peak at 3336 cm^−1^ from a broad peak was more prominent from NCC-1 to NCC-3, showing that the cellulose changed with various hydrogen bonding interactions [33]. The peak at 2899 cm^−1^ is associated with the C–H bending, indicating the improved exposure of the cellulose components that became sharper for samples with longer hydrolysis time [13].

The absence of peaks at 1662 cm^−1^, 1548 cm^−1^, and 1249 cm^−1^ in all the NCC samples was due to the removal of hemicellulose and lignin by bleaching. The small visible peak at 1736 cm^−1^ was generated by C=O stretching of non-conjugated ketone, carbonyl, and ester groups. The weakness of this peak can be attributed to the weakness of the connection of lignin and hemicellulose [34]. Furthermore, the peaks at 1056 cm^−1^ and 891 cm^−1^ can be assigned to the C–OH stretching and C–O–C symmetric glycosidic bonds, respectively, during NCC synthesis. Both peaks indicated the cellulose crystalline content and structure detailed elsewhere [4,11,35]. The peak at 850 cm^−1^ is associated with the C–O–S bending vibration in C–O–SO^3−^ groups, indicating the NCC sulfation peak, and was detected in NCC-2 and NCC-3 but not significantly in NCC-1. This could be attributed to the prolonged hydrolysis time, in which sulfuric acid had a longer reaction time with the PCLF and remained in the NCC [36,37]. This study shows that the morphological structure of the NCC samples was intact, and the increase in peak intensity was due to the increase in cellulose crystallinity.

#### 3.2.2. Crystalline Structures

**Figure 3:**
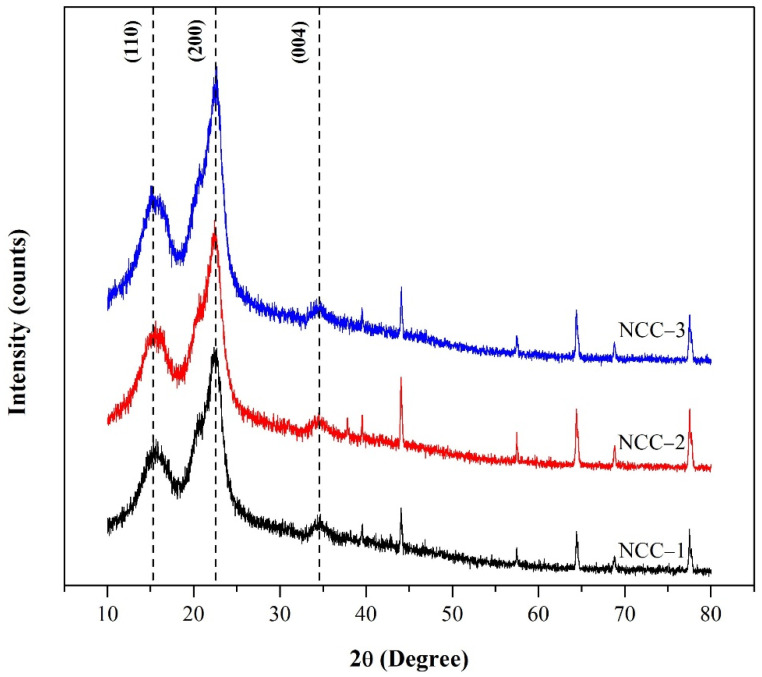
XRD pattern of nanocrystalline cellulose from pineapple crown leaf fibers with various hydrolysis times.

The XRD diffractogram obtained for the NCC samples from the PCLF with various hydrolysis times is shown in Figure 3. All the NCC samples showed diffraction peak at 16°, 22.5°, and 34.4°, correlating to the (110), (200), and (004) crystal planes of the Iβ cellulose structure [38,39]. The diffraction peak at 22.5° was significantly enhanced in the NCC-2 and NCC-3 spectra, indicating the cellulose crystalline regions were greater in the samples [11,30].

The crystallinity value and crystal size of NCC from PCLF are shown in Table 3. The increase in the crystallinity value of the NCC samples prepared under a longer hydrolysis time was caused by the amorphous regions containing the crystalline parts, which dissolved during acid hydrolysis and released more individual crystals [30,40]. The crystallinity in this study was higher than the crystallinity of cellulose from *Typha* sp. [40]. Furthermore, the crystal sizes were found to lower with increasing hydrolysis time. The decrease in the crystallite size of NCC could be attributed to the process of acid reaction breaking into the cellulose layer and hydrolyzing the smaller cellulose crystals [40]. The removal of the amorphous region increased the crystallinity and lowered the NCC crystallite sizes.

#### 3.2.3. Surface Morphology

**Figure 4:**
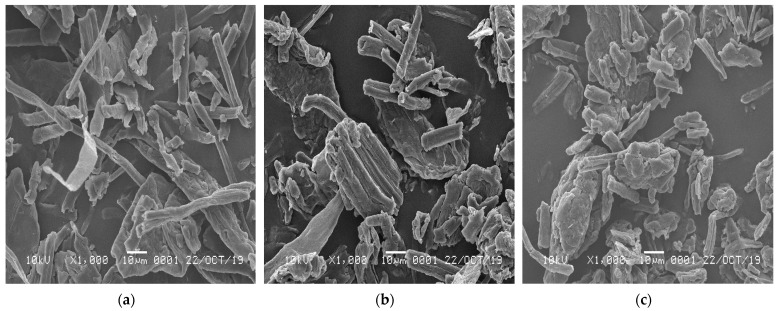
SEM micrographs of (**a**) nanocrystalline cellulose (NCC)-1; (**b**) NCC-2; and (**c**) NCC-3 under 1000× magnification.

The micrographs of all the NCC samples showing their morphology are shown in Figure 4. The SEM micrograph of the NCC-1 sample shows it had rod-like features with a rough surface. Such a morphology likely originated from the undisintegrated fibrous structure that remained intact during one hour of hydrolysis. Increasing the hydrolysis time to two and three hours showed a short nanocrystalline particle formation in the NCC-2 and NCC-3 samples. The disintegration process of the fibers seemed to begin at this stage, as reported earlier [11,30].

Furthermore, the NCC-3 sample presented short and individually segregated nanoparticles. These results are consistent with the result obtained from the FTIR analysis. Sulfuric acid with longer reaction times caused the NCC to disintegrate into shorter crystals and reduced the cellulose chain hydrogen bonding, resulting in smaller NCC particles [16]. The reduction of the NCC size may also be due to the amorphous segment removals that otherwise surrounded the fibrils in the cellulose matrix [30]. The longer reaction time of H_2_SO_4_ on the fibers via the hydrolysis process removed the amorphous part of the PCLF, resulting in a smaller NCC. However, this depends on the hydrolysis process conditions and the variety of cellulosic fiber sources [41]. A similar result was obtained in a previous study using sugar palm fiber and kenaf bast fibers with hydrolysis for 30, 45, and 60 min [36].

**Figure 5:**
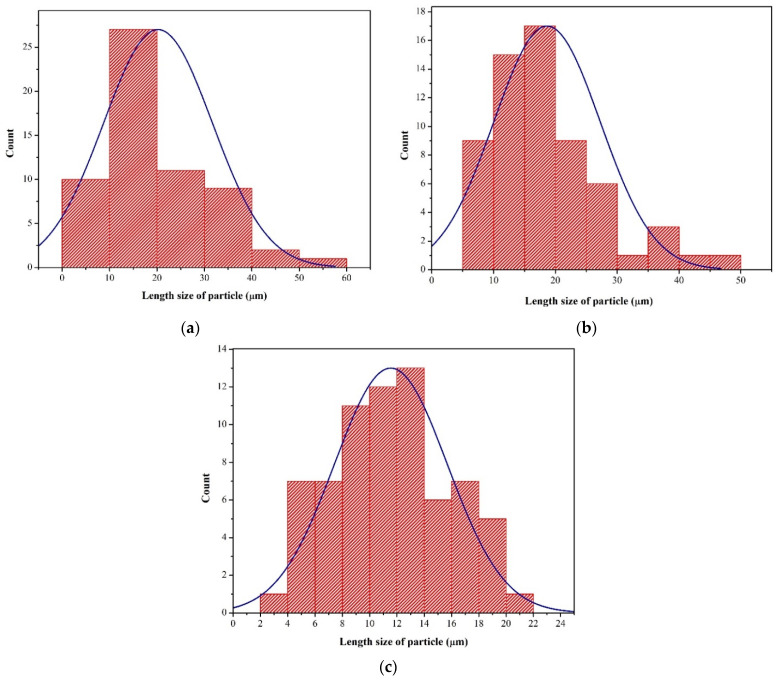
Length size distribution of (**a**) nanocrystalline cellulose (NCC)-1; (**b**) NCC-2; and (**c**) NCC-3.

From the SEM extended analysis, the shapes and lengths of all the NCC samples show standard distributions (Figure 5). The estimated average lengths for NCC-1, NCC-2, and NCC-3 were 20.21, 18.64, and 11.55 µm, respectively. The average length size particle of the NCC samples decreased with increasing hydrolysis time. The noticeable reduction in the length of the NCC particles was caused by the removal of more amorphous regions that surrounded the cellulose and the disintegration of the crystalline domains [4]. This finding was also consistent with the crystal size analysis, where the increase in hydrolysis time resulted in a shorter crystal length of the NCC. The sulfuric acid could also break down the glycosidic bonds in NCC and release more shorter-chained oligomers [30].

#### 3.2.4. Thermal Analysis

The thermal stability of NCC materials is crucial to estimate their performance and use in several applications, especially as a reinforcing agent of nanocomposite materials. The TGA and DTG results of the NCC formed from various hydrolysis durations are shown in Table 3 and Figure 4. The curves of cellulose show three typical stages of weight loss. In the first stage, each sample showed a slight weight loss in the range of 25–130 °C, with an initial weight loss of nearly 7% because of the evaporation of the remaining bond- water and other volatile matters. In the second stage, the mass reduction was observed in a temperature range of 131–400 °C. In this stage, the NCC chain and chemical bonds were broken, which led to the dehydration, depolymerization, and decomposition of NCC [42,43]. The last stage was NCC decomposition, which occurred in the temperature range of 400–500 °C. It was attributed to the residual material deformation.

**Figure 6:**
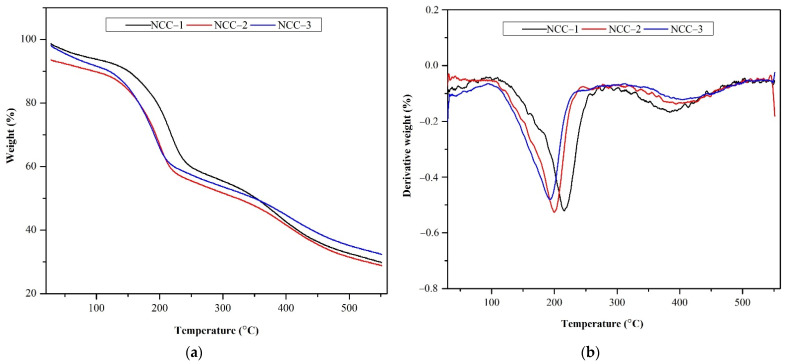
(**a**) TGA and (**b**) DTG spectra of nanocrystalline cellulose with various hydrolysis times.

As shown in Table 4 and Figure 6, the NCC samples experienced slight differences in thermal stabilities. The onset decomposition temperatures of NCC-1, NCC-2, and NCC-3 were 176.98, 157.05, and 149.03 °C, respectively. The decrease in the onset decomposition temperature of the NCC could have occurred because of the treatment of cellulose with sulfuric acid, leading to a visible decrease in the thermal properties of the formed NCC. The reduction of the decomposition temperature occurred because of the incorporation of the sulfate groups on the surface of the NCC after the hydrolysis process, as reported earlier [4]. The presence of the acid sulfate group has a catalytic impact on the thermal degradation process and reduces the thermal stability of NCC as a result of the dehydration reaction [44,45].

Furthermore, the acid hydrolysis resulted in a significant decrease in the thermal stability of NCC; it was predicted that the beginning decomposition temperature of NCC-2 would be lower than that of NCC-1. This can be explained by the higher cellulose crystallite content of NCC-2 compared to NCC-1, the short hydrolysis period, and the low sulfate group content [46]. This study found a similar result with NCC-3, which had a significantly greater sulfate content than NCC-2. This finding is consistent with the results of the FTIR, XRD, and SEM measurements.

The maximum decomposition temperatures of the NCC samples obtained from the DTG data were at 215.88, 195.82, and 186.90 °C for NCC-1, NCC-2, and NCC-3, respectively. The reduction in maximum temperature can be ascribed to the creation of a short and free end-chain of NCC particles that can be degraded at lower temperatures and the destruction of some crystalline parts of the cellulose from PCLF over a longer hydrolysis time, as discussed earlier. Similar results were also reported by Aprilia et al. [30], Mohamad Haafiz et al. [47], and Kian et al. [48] for NCC extracted from kenaf bast, oil palm biomass, and roselle fiber, respectively. In addition, NCC-1 also had the highest peak of decomposition temperature at 215.88 °C, probably because of the arrangement of the resulting NCC in comparison to the ones formed under longer hydrolysis times [13]. The lower sulfate group incorporation in NCC-1 resulted in a more compact cellulose crystal and an undisintegrated fibrous structure, resulting in a higher thermal stability [49].

Meanwhile, the NCC-3 showed a higher char formation residue compared to that of NCC-1 and NCC-2. It was related to the high flame resistance of the cellulose crystals in the samples [16]. In the hydrolysis process, sulfuric acid is a well-known dehydration catalyst that facilitates char residue formation [50,51,52]. The lower weight loss in the NCC also demonstrated the capability of the cellulose structure to tolerate high temperatures [53] and widen its high-temperature stability.

## 4. Conclusions

This study reports the application of sulfuric acid for isolating NCC products from PCLF and investigates the effect of hydrolysis times on the resulting NCC properties. Short and rod-like particle shapes were produced under longer hydrolysis times, as shown by the SEM images. Furthermore, the hydrolysis condition had a significant influence on the functional group for all samples. The acid hydrolysis removed the non-cellulosic components, resulting in low lignin but a high cellulose content. It was possible to obtain stable aqueous suspensions of NCC, which were negatively charged because of sulfate groups for the NCC-3 sample. The crystallinity of the NCC increased to 63.34% by removing amorphous regions during hydrolysis, which also reduced the crystal size of the NCC to 17.99 nm. The yield of the NCC product was the highest at 79.37% at one hour of hydrolysis, higher than the yield from rice hull and bean hull fibers. The NCC-1 was the most thermally stable, which can be advantageous when used as a filler application. The overall findings suggest that isolating NCC from PCLF shows exciting properties that can be used in several applications with high-added value and gives a promising method for developing NCC as a potential reinforcement agent in composite materials. In addition, the reuse of this agricultural waste allows for a significant reduction and an essential contribution to solving the agricultural disposal problem.

## Figures and Tables

**Table 1:** Lignocellulosic composition of pineapple crown leaf fibers.

Source	Hot Water Soluble	Hemicellulose	Cellulose	Lignin	Ash
Pineapple crown leaf	20.80	13.30	51.20	13.40	2.30
Pineapple leaf ^a^	-	20.4	74.50	8.72	2.28
Pineapple leaf fibers ^b^	-	12.31	81.27	3.46	-
Pineapple crown fibers ^c^	14.1	16.80	56.00	13.10	-

^a^ Santos et al. [4]; ^b^ Cherian et al. [25]; ^c^ Mamani et al. [26].

**Table 2:** Yield nanocrystalline cellulose from pineapple crown leaf fibers.

Samples	Yield (%)
NCC-1	79.37 ± 1.46
NCC-2	78.10 ± 1.18
NCC-3	76.23 ± 1.74

Results expressed as mean ± standard deviation.

**Table 3:** Crystallinity index and crystallite size of nanocrystalline cellulose from pineapple crown leaf fibers.

Samples	Crystallinity Index (%)	Crystal Size (nm)
NCC-1	54.92	21.00
NCC-2	58.46	19.90
NCC-3	63.34	17.99

**Table 4:** Thermal property parameters of nanocrystalline cellulose from pineapple crown leaf fibers.

Samples	T_onset_ (°C) ^a^	T_max_ (°C) ^b^	Residual Weight (%) ^c^
NCC-1	176.98	215.88	30.38
NCC-2	157.05	195.82	29.06
NCC-3	149.03	186.90	32.98

^a^ TGA onset decomposition temperature; ^b^ DTG maximum temperature; ^c^ residual weight formation at 550 °C.

## Data Availability

The data presented in this study are available on request from the corresponding author.

## References

[1] Prado K.S., Spinace M.A.S. (2019). Isolation and characterization of cellulose nanocrystals from pineapple crown waste and their potential uses. Int. J. Biol. Macromol..

[2] Tran A.V. (2006). Chemical analysis and pulping study of pineapple crown leaves. Ind. Crop. Prod..

[3] Brinchi L., Cotana F., Fortunati E., Kenny J.M. (2013). Production of nanocrystalline cellulose from lignocellulosic biomass: Technology and applications. Carbohydr. Polym..

[4] Dos Santos R.M., Flauzino Neto W.P., Silvério H.A., Martins D.F., Dantas N.O., Pasquini D. (2013). Cellulose nanocrystals from pineapple leaf, a new approach for the reuse of this agro-waste. Ind. Crop. Prod..

[5] Mukherjee P.S., Satyanarayana K.G. (1984). Structure and properties of some vegetable fibres. J. Mater. Sci..

[6] Sharma U.S. (1981). Investigations on the fibers of pineapple [*Ananas comosus* (L). Merr.] leaves. Carbohydr. Res..

[7] Asim M., Abdan K., Jawaid M., Nasir M., Dashtizadeh Z., Ishak M.R., Hoque M.E. (2015). A Review on Pineapple Leaves Fibre and Its Composites. Int. J. Polym. Sci..

[8] Saha S.C., Das B.K., Ray P.K., Pandey S.N., Goswami K. (1990). SEM Studies of the Surface and Fracture Morphology of Pineapple Leaf Fibers. Text. Res. J..

[9] Azubuike C.P., Okhamafe A.O. (2012). Physicochemical, spectroscopic and thermal properties of microcrystalline cellulose derived from corn cobs. Int. J. Recycl. Org. Waste Agric..

[10] Frone A.N., Panaitescu D.M., Donescu D. (2011). Some Aspects Concerning The Isolation of Cellulose Micro and Nanofibers. UPB Sci. Bull. Ser. B Chem. Mater. Sci..

[11] Khan A., Jawaid M., Kian L.K., Khan A.A.P., Asiri A.M. (2021). Isolation and Production of Nanocrystalline Cellulose from Conocarpus Fiber. Polymers.

[12] Alharthi S., Grishkewich N., Berry R.M., Tam K.C. (2020). Functional cellulose nanocrystals containing cationic and thermo-responsive polymer brushes. Carbohydr. Polym..

[13] Xing L., Gu J., Zhang W., Tu D., Hu C. (2018). Cellulose I and II nanocrystals produced by sulfuric acid hydrolysis of Tetra pak cellulose I. Carbohydr. Polym..

[14] Pirich C.L., Picheth G.F., Machado J.P.E., Sakakibara C.N., Martin A.A., de Freitas R.A., Sierakowski M.R. (2019). Influence of mechanical pretreatment to isolate cellulose nanocrystals by sulfuric acid hydrolysis. Int. J. Biol. Macromol..

[15] Sutliff B.P., Das A., Youngblood J., Bortner M.J. (2020). High shear capillary rheometry of cellulose nanocrystals for industrially relevant processing. Carbohydr. Polym..

[16] Alothman O.Y., Kian L.K., Saba N., Jawaid M., Khiari R. (2021). Cellulose nanocrystal extracted from date palm fibre: Morphological, structural and thermal properties. Ind. Crop. Prod..

[17] Alizadeh-Sani M., Khezerlou A., Ehsani A. (2018). Fabrication and characterization of the bionanocomposite film based on whey protein biopolymer loaded with TiO_2_ nanoparticles, cellulose nanofibers and rosemary essential oil. Ind. Crop. Prod..

[18] Kuthi F.A.A., Norzali N.R.a.A., Badri K.H. (2016). Thermal Characteristics of Microcrystalline Cellulose from Oil Palm Biomass. Malays. J. Anal. Sci..

[19] Fitriani, Aprilia N.A.S., Arahman N. (2020). Properties of nanocrystalline cellulose from pineapple crown leaf waste. IOP Conf. Ser. Mater. Sci. Eng..

[20] Chesson A.L. (1978). The Maceration of Linen Flax under Anaerobic Conditions. J. Appl. Microbiol..

[21] Datta R. (1981). Acidogenic fermentation of lignocellulose acid yield and conversion of components. Biotechnol. Bioeng..

[22] French A.D., Santiago Cintrón M. (2013). Cellulose polymorphy, crystallite size, and the Segal Crystallinity Index. Cellulose.

[23] Segal L., Creely J.J., Martin A.E., Conrad C.M. (1959). An Empirical Method for Estimating the Degree of Crystallinity of Native Cellulose Using the X-Ray Diffractometer. Text. Res. J..

[24] Jin X.-J., Pascal Kamdem D. (2009). Chemical composition, crystallinity and crystallite cellulose size in populus hybrids and aspen. Cellul. Chem. Technol..

[25] Cherian B.M., Leão A.L., de Souza S.F., Thomas S., Pothan L.A., Kottaisamy M. (2010). Isolation of nanocellulose from pineapple leaf fibres by steam explosion. Carbohydr. Polym..

[26] Choquecahua Mamani D., Otero Nole K.S., Chaparro Montoya E.E., Mayta Huiza D.A., Pastrana Alta R.Y., Aguilar Vitorino H. (2020). Minimizing Organic Waste Generated by Pineapple Crown: A Simple Process to Obtain Cellulose for the Preparation of Recyclable Containers. Recycling.

[27] Asghari M., Karimi Zarchi A.A., Taheri R.A. (2021). Preparation and Characterization Nanocrystalline Cellulose as a Food Additive to Produce Healthy Biscuit Cream. Starch-Stärke.

[28] Sartika D., Syamsu K., Warsiki E., Fahma F., Arnata I.W. (2021). Nanocrystalline Cellulose from Kapok Fiber (*Ceiba pentandra*) and its Reinforcement Effect on Alginate Hydrogel Bead. Starch-Stärke.

[29] Guo J., Zhuang Y., Chen L., Liu J., Li D., Ye N. (2012). Process optimization for microwave-assisted direct liquefaction of *Sargassum polycystum* C.Agardh using response surface methodology. Bioresour. Technol..

[30] Aprilia N.A.S., Davoudpour Y., Zulqarnain W., Khalil H.A., Hazwan C.M., Hossain M.S., Dungani R., Fizree H.M., Zaidon A., Haafiz M.K.M. (2016). Physicochemical Characterization of Microcrystalline Cellulose Extracted from Kenaf Bast. Bioresources.

[31] Adel A.M., Abd El-Wahab Z.H., Ibrahim A.A., Al-Shemy M.T. (2010). Characterization of microcrystalline cellulose prepared from lignocellulosic materials. Part I. Acid catalyzed hydrolysis. Bioresour. Technol..

[32] Singh S., Gaikwad K.K., Park S.I., Lee Y.S. (2017). Microwave-assisted step reduced extraction of seaweed (*Gelidiella aceroso*) cellulose nanocrystals. Int. J. Biol. Macromol..

[33] Du W., Deng A., Guo J., Chen J., Li H., Gao Y. (2019). An injectable self-healing hydrogel-cellulose nanocrystals conjugate with excellent mechanical strength and good biocompatibility. Carbohydr. Polym..

[34] Kejun S., Juntao Z., Ying C., Zongwen L., Lin R., Cong L. (2011). Accelerating the degradation of green plant waste with chemical decomposition agents. J. Environ. Manag..

[35] Luzi F., Fortunati E., Puglia D., Lavorgna M., Santulli C., Kenny J.M., Torre L. (2014). Optimized extraction of cellulose nanocrystals from pristine and carded hemp fibres. Ind. Crop. Prod..

[36] Ilyas R.A., Sapuan S.M., Atikah M.S.N., Asyraf M.R.M., Rafiqah S.A., Aisyah H.A., Nurazzi N.M., Norrrahim M.N.F. (2020). Effect of hydrolysis time on the morphological, physical, chemical, and thermal behavior of sugar palm nanocrystalline cellulose (*Arenga pinnata* (Wurmb.) Merr). Text. Res. J..

[37] Vasconcelos N.F., Feitosa J.P., da Gama F.M., Morais J.P., Andrade F.K., de Souza Filho M.S., Rosa M.F. (2017). Bacterial cellulose nanocrystals produced under different hydrolysis conditions: Properties and morphological features. Carbohydr. Polym..

[38] Seta F.T., An X., Liu L., Zhang H., Yang J., Zhang W., Nie S., Yao S., Cao H., Xu Q. (2020). Preparation and characterization of high yield cellulose nanocrystals (CNC) derived from ball mill pretreatment and maleic acid hydrolysis. Carbohydr. Polym..

[39] Neto W.P.F., Silvério H.A., Dantas N.O., Pasquini D. (2013). Extraction and characterization of cellulose nanocrystals from agro-industrial residue—Soy hulls. Ind. Crop. Prod..

[40] Rahmawati C., Aprilia S., Saidi T., Aulia T.B., Ahmad I. (2021). Preparation and Characterization of Cellulose Nanocrystals from *Typha* sp. as a Reinforcing Agent. J. Nat. Fibers.

[41] Chan H.C., Chia C.H., Zakaria S., Ahmad I.S.K., Dufresne A. (2012). Production and Characterisation of Cellulose and Nano-Crystalline Cellulose from Kenaf Core Wood. Bioresources.

[42] Jiang Y., Zhou J., Zhang Q., Zhao G., Heng L., Chen D., Liu D. (2017). Preparation of cellulose nanocrystals from Humulus japonicus stem and the influence of high temperature pretreatment. Carbohydr. Polym..

[43] Li M., He B., Chen Y., Zhao L. (2021). Physicochemical Properties of Nanocellulose Isolated from Cotton Stalk Waste. ACS Omega.

[44] Roman M., Winter W.T. (2004). Effect of sulfate groups from sulfuric acid hydrolysis on the thermal degradation behavior of bacterial cellulose. Biomacromolecules.

[45] Fahma F., Iwamoto S., Hori N., Iwata T., Takemura A. (2010). Isolation, preparation, and characterization of nanofibers from oil palm empty-fruit-bunch (OPEFB). Cellulose.

[46] Chen Y., Liu C., Chang P.R., Anderson D.P., Huneault M.A. (2009). Pea starch-based composite films with pea hull fibers and pea hull fiber-derived nanowhiskers. Polym. Eng. Sci..

[47] Mohamad Haafiz M.K., Eichhorn S.J., Hassan A., Jawaid M. (2013). Isolation and characterization of microcrystalline cellulose from oil palm biomass residue. Carbohydr. Polym..

[48] Kian L.K., Jawaid M. (2019). Thermal Properties of Nanocrystalline Cellulose and Cellulose Nanowhisker. Int. J. Innov. Technol. Explor. Eng..

[49] Kargarzadeh H., Ahmad I., Abdullah I., Dufresne A., Zainudin S.Y., Sheltami R.M. (2012). Effects of hydrolysis conditions on the morphology, crystallinity, and thermal stability of cellulose nanocrystals extracted from kenaf bast fibers. Cellulose.

[50] Kim D.-Y., Nishiyama Y., Wada M., Kuga S. (2001). High-yield Carbonization of Cellulose by Sulfuric Acid Impregnation. Cellulose.

[51] Rosa M.F., Medeiros E.S., Malmonge J.A., Gregorski K.S., Wood D.F., Mattoso L.H.C., Glenn G., Orts W.J., Imam S.H. (2010). Cellulose nanowhiskers from coconut husk fibers: Effect of preparation conditions on their thermal and morphological behavior. Carbohydr. Polym..

[52] Wang N., Ding E., Cheng R. (2007). Thermal degradation behaviors of spherical cellulose nanocrystals with sulfate groups. Polymer.

[53] Bano S., Negi Y.S. (2017). Studies on cellulose nanocrystals isolated from groundnut shells. Carbohydr. Polym..

